# Supplementary material for: The causal role of circulating inflammatory markers in osteoporosis: a bidirectional Mendelian randomized study
Source: Front Immunol. 2024 Jul 18;15:1412298. doi: 10.3389/fimmu.2024.1412298 (PMC11291241; doi:10.3389/fimmu.2024.1412298)
Supplement: Supplementary file 1 [file DataSheet_1.zip › supplementary material/OSTEOPOROSIS-CIM/GCST90274834.tsv.gz_leave_one_out.pdf]

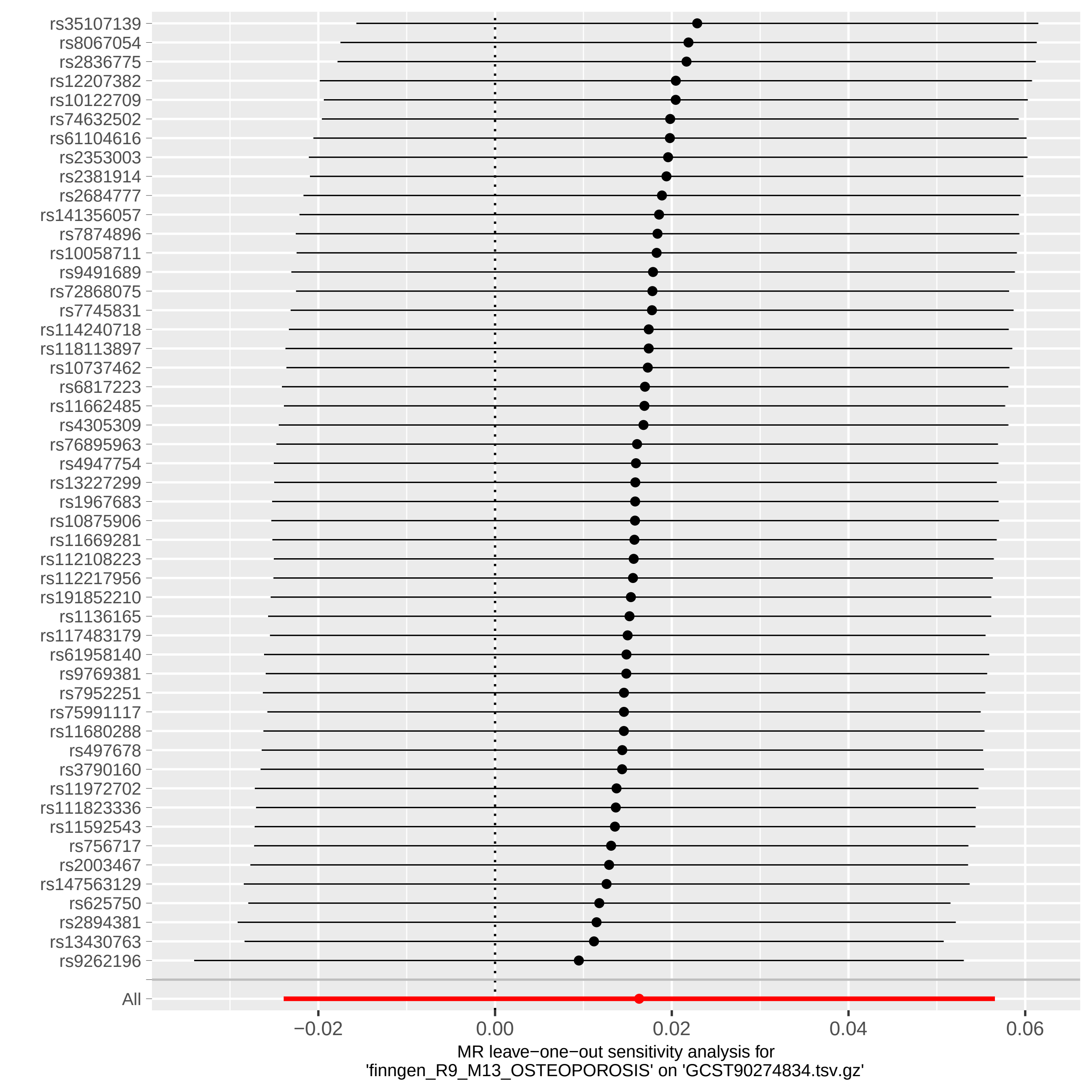

rs35107139
rs8067054
rs2836775
rs12207382
rs10122709
rs74632502
rs61104616
rs2353003
rs2381914
rs2684777
rs141356057
rs7874896
rs10058711
rs9491689
rs72868075
rs7745831
rs114240718
rs118113897
rs10737462
rs6817223
rs11662485
rs4305309
rs76895963
rs4947754
rs13227299
rs1967683
rs10875906
rs11669281
rs112108223
rs112217956
rs191852210
rs1136165
rs117483179
rs61958140
rs9769381
rs7952251
rs75991117
rs11680288
rs497678
rs3790160
rs11972702
rs111823336
rs11592543
rs756717
rs2003467
rs147563129
rs625750
rs2894381
rs13430763
rs9262196
All
−0.02
0.00
0.02
0.04
0.06
MR leave−one−out sensitivity analysis for
'finngen_R9_M13_OSTEOPOROSIS' on 'GCST90274834.tsv.gz'
